# Elevated levels of alanine transaminase and triglycerides within normal limits are associated with fatty liver

**DOI:** 10.3892/etm.2014.1798

**Published:** 2014-06-23

**Authors:** MINORU TOMIZAWA, YUJI KAWANABE, FUMINOBU SHINOZAKI, SUMIHIKO SATO, YASUFUMI MOTOYOSHI, TAKAO SUGIYAMA, SHIGENORI YAMAMOTO, MAKOTO SUEISHI

**Affiliations:** 1 Department of Gastroenterology, National Hospital Organization Shimoshizu Hospital, Yotsukaidō, Chiba 284-0003, Japan; 2 Sato Clinic, Wakaba-ku, Chiba, Chiba 264-0021, Japan; 3 Department of Radiology, National Hospital Organization Shimoshizu Hospital, Yotsukaidō, Chiba 284-0003, Japan; 4 Department of Neurology, National Hospital Organization Shimoshizu Hospital, Yotsukaidō, Chiba 284-0003, Japan; 5 Department of Rheumatology, National Hospital Organization Shimoshizu Hospital, Yotsukaidō, Chiba 284-0003, Japan; 6 Department of Pediatrics, National Hospital Organization Shimoshizu Hospital, Yotsukaidō, Chiba 284-0003, Japan

**Keywords:** receiver operating characteristics, threshold

## Abstract

In the present study, the threshold values of laboratory data for the diagnosis of non-alcoholic fatty liver disease (NAFLD) were investigated. The study enrolled patients who had undergone abdominal ultrasound (US) between April 2013 and August 2013, and for whom laboratory data were available on the same day. NAFLD was diagnosed following observations of a bright liver or hepatorenal echo contrast on the abdominal US scans. Patients were excluded from the study if they had liver diseases or had been prescribed prednisolone or methotrexate. Receiver operating characteristic curves, the Wilcoxon signed-rank test and Fisher’s exact probability test were used for data analysis. In total, 80 NAFLD and 94 non-NAFLD patients were enrolled in the study. The threshold levels of alanine aminotransferase (ALT) and triglyceride (TG) for the diagnosis of NAFLD were 19.0 IU/l and 101 mg/dl, respectively. Patients were divided into two groups according to the levels of ALT and TG. Those with ALT levels of >19 IU/l and TG levels of >101 mg/dl were defined as the positive group, while the remaining patients were classified as the negative group. The specificity and positive predictive value using the combined threshold levels of ALT >19 IU/l and TG >101 mg/dl were 80.9 and 75.0%, respectively. Therefore, the results indicated that ALT levels of >19 IU/l or TG levels of >101 mg/dl were useful markers for the screening of NAFLD. However, NAFLD was more strongly suspected in patients with ALT levels of >19 IU/l and TG levels of >101 mg/dl.

## Introduction

Non-alcoholic fatty liver disease (NAFLD) is defined as the presence of fat accumulation in the liver, detected by imaging or histology, with no causes of secondary fat accumulation, including significant alcohol consumption (1,2). NAFLD is associated with obesity, diabetes and hyperlipidemia, and can be subcategorized into non-alcoholic fatty liver and non-alcoholic steatohepatitis (NASH). NASH is differentiated by the presence of hepatocyte injury (1). The survival rate of NAFLD patients is lower than that of the general population standardized mortality ratio resulting from cardiovascular disease and hepatocellular carcinoma (3–5). Thus, it is important for NAFLD to be diagnosed and treated (6). The diagnosis of NAFLD is based on an assessment of fat accumulation in the liver by imaging or liver biopsy. Abdominal ultrasound (US) is the least complicated of the diagnostic imaging modalities, which include magnetic resonance imaging and computed tomography (7). Elastography, an advanced form of abdominal US, is a new method of diagnosing NASH by evaluating fibrosis (8). One of the limitations of elastography is that the examination is expensive and not widely available. Liver biopsy is the most accurate diagnostic method and is considered the gold standard, but harbors limitations due to the invasiveness of the technique. Abdominal US is the first-line test for fat accumulation in the liver (9). However, it is not practical to perform abdominal US in all patients to screen for NAFLD. The ability to triage patients with suspected NAFLD and selectively perform abdominal US is desirable. The thresholds of waist circumference for the diagnosis of NAFLD are 85.0 cm in males and 80.0 cm in females (2). However, despite the determination of waist circumference being simple and requiring no equipment, the examination is not accurate due to operator dependency.

Blood examinations are performed widely, and laboratory data are quantitative and reliable. However, laboratory data with regard to NAFLD remain controversial. NAFLD patients have been shown to have higher levels of alkaline phosphatase (ALP), aspartate aminotransferase (AST), alanine aminotransferase (ALT) and γ-glutamyl transpeptidase (γ-GTP) (10). By contrast, Chalasani *et al* reported that laboratory data of patients with NAFLD and NASH can be within the normal ranges (1).

Therefore, in the present study, the association between laboratory data and NAFLD was investigated with the aim of identifying thresholds for the diagnosis of NAFLD.

## Materials and methods

### Inclusion criteria

Patients that had undergone abdominal US between April 2013 and August 2013, and that had laboratory data available on the date of abdominal US, were enrolled in the study. Patient records were analyzed retrospectively. Patients were divided into two groups: Non-NAFLD patients (NF; n=94) and NAFLD patients (F; n=80). The study protocol was submitted to the Institutional Ethical Committee of the National Hospital Organization Shimoshizu Hospital (Yotsukaidō, Japan), and the study was determined to not be a clinical trial since it was performed as part of routine clinical practice. Written informed patient consent was obtained from the patient/ or the patient’s family. Patient anonymity was preserved throughout the study.

### Exclusion criteria

Patients were excluded from the study if laboratory data from the day of the US were not available. Patients were also excluded if they tested positive for the hepatitis B virus surface antigen or anti-hepatitis C virus antibody. The presence of liver cirrhosis, primary biliary cirrhosis, autoimmune hepatitis or high alcohol consumption also excluded patients from the study due to the potentially elevated liver enzymes (11,12). In addition, patients with muscular dystrophy or dermatomyositis were excluded due to the potentially elevated AST or lactate dehydrogenase (LDH) levels. Patients were also excluded if they had been prescribed prednisolone, which can cause NAFLD (13), or if they had been prescribed methotrexate due to the potential of this drug in inducing liver toxicity (14).

### Abdominal US

Diagnosis of NAFLD was determined using abdominal US with standardized criteria (15,16). Briefly, NAFLD was diagnosed when a bright liver or hepatorenal echo contrast was observed on the abdominal US scans. Abdominal US was performed by Senior Fellows of the Japan Society of Ultrasonics in Medicine with an SSA-700A instrument (Toshiba Medical Systems Corporation, Ohtawara, Japan) using a 3.75 MHz curved-array probe (PVT-375BT; Toshiba Medical Systems Corporation) in the US unit. Abdominal US was performed by Board Certified Fellows of the Japan Society of Ultrasonics in Medicine. Operators were blinded to the clinical and laboratory data.

### Laboratory data

Analyzed laboratory parameters included ALP, AST, ALT, γ-GTP, LDH, high-density lipoprotein cholesterol (HDL), low-density lipoprotein cholesterol (LDL), triglycerides (TG) and total cholesterol (T-chol) levels.

### Statistical analysis

Receiver operating characteristic (ROC) curves were created using JMP 10.0.2 software (SAS Institute, Cary, NC, USA). Parameters, including the patient age and the levels of ALP, AST, ALT, γ-GTP, LDH, HDL, LDL, TG and T-chol, were investigated on the day of abdominal US. The area under the curve (AUC) was used as a measure of diagnostic efficacy. The threshold value was determined as the highest sensitivity and specificity values, and was calculated automatically using software that determined the location where a line with a slope of 45° contacted the ROC curve. The Wilcoxon signed-rank test was used for the comparison of variables between the NF and F groups. In addition, Fisher’s exact probability test was used to compare the sensitivity of using ALT levels of >19 IU/l and TG levels of >101 mg/dl for the diagnosis of NAFLD. P<0.05 was considered to indicate a statistically significant difference.

## Results

### Laboratory data

A total of 80 NAFLD and 94 non-NAFLD patients were enrolled in the study. The laboratory data of each group are presented in Table I. Levels of AST, ALT, HDL, LDL, TG and T-chol were higher in the F group compared with the NF group.

### ROC analysis

**Figure 1:**
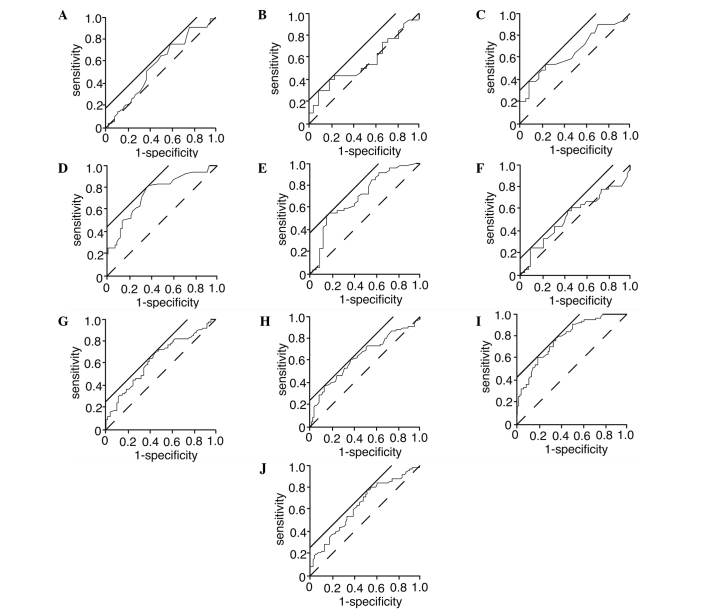
ROC curves of the results from the blood examinations conducted on the day of the abdominal US. Analyzed data included (A) patient age and levels of (B) ALP, (C) AST, (D) ALT, (E) γ-GTP, (F) LDH, (G) HDL, (H) LDL, (I) TG and (J) T-chol. The solid straight line (with a slope of 45°) was used to calculate the threshold value using JMP 8.0.2 software. The broken line was used as a reference. ROC, receiver operating characteristic; US, ultrasound; ALP, alkaline phosphatase; AST, aspartate aminotransferase; ALT, alanine aminotransferase; γ-GTP, γ-glutamyl transpeptidase; LDH, lactate dehydrogenase; HDL, high-density lipoprotein cholesterol; LDL, low-density lipoprotein cholesterol; TG, triglycerides; T-chol, total cholesterol.

Fig. 1 shows the ROC analysis of the variables for the diagnosis of NAFLD. The AUC, sensitivity and specificity values of each variable are presented in Table II. The AUC for ALT was 0.743, and the threshold of ALT for the diagnosis of NAFLD was 19.0 IU/l. Sensitivity and specificity values at this threshold were 80.0 and 63.5%, respectively. The AUC for TG was 0.778, and the threshold of TG for the diagnosis of NAFLD was 101 mg/dl. Sensitivity and specificity values at this threshold were 78.4 and 64.3%, respectively. The sensitivities of these two variables were ~80%, however, the specificity values were <65%, which was low.

To improve the diagnostic accuracy, a combination of the thresholds of ALT and TG was analyzed for the diagnosis of NAFLD. Patients were divided into two groups according to the levels of ALT and TG. Patients with ALT levels of >19 IU/l and TG levels of >101 mg/dl were categorized as ‘positive’, while the remaining patients were categorized as ‘negative’. Table III shows a two-contingency table according to the diagnosis of fatty liver or non-fatty liver with the combined threshold value of ALT >19 IU/l and TG >101 mg/dl. For this threshold combination, the sensitivity, specificity, positive predictive value and negative predictive value were 67.5% [95% confidence interval (CI), 60.0–73.7], 80.9% (95% CI, 74.5–86.2), 75.0% (95% CI, 66.7–81.9) and 74.5% (95% CI, 68.7–79.4), respectively. Each value was calculated based on Table III.

## Discussion

Elevated ALT levels are associated with NAFLD clinically and histologically (10,17). The levels of ALT reflect the eating habits of the patient, with decreased levels observed following the consumption of a diet high in vegetables and low in animal-based protein (18). In the present study, the threshold value of ALT for the diagnosis of NAFLD was 19.0 IU/l. Notably, the threshold value was within the normal limits. In the study by Wu *et al*, the upper normal limit of ALT was analyzed (19). The authors enrolled 34,346 subjects who completed a health check-up, and excluded subjects with risk factors associated with elevated ALT levels, including high body mass index, high waist circumference, high glucose levels, high cholesterol levels, low levels of HDL, high levels of TG, hepatitis B virus surface antigen, anti-hepatitis C virus antibody and NAFLD. The threshold of ALT selected in the present study was within the normal limit of 27 IU/l. It has been hypothesized that a slight elevation in the levels of ALT is indicative of NAFLD (20). This hypothesis is supported by previous studies that have reported that a slight elevation in the levels of ALT and γ-GTP within the normal limits indicates NAFLD (21,22).

TG levels are more commonly associated with NAFLD, as compared with LDL and HDL levels (23). In the present study, TG consistently exhibited the highest AUC. In addition, the present study found that the threshold of TG for the diagnosis of NAFLD was 101 mg/dl. To the best of our knowledge, these results are the first with regard to the threshold of TG. The observations of the current study clearly demonstrate that ALT and TG levels were useful for the diagnosis of NAFLD, as reported previously (24).

In the present study, the individual thresholds of ALT and TG for the diagnosis of NAFLD exhibited low specificity. Thus, a combination of ALT and TG thresholds was investigated with the aim of improving the NAFLD diagnostic capability. The specificity and positive predictive value, when using the combined thresholds of ALT levels of >19 IU/l and TG levels of >101 mg/dl, were 80.9 and 75.0%, respectively. Therefore, using the combination of ALT and TG thresholds was useful for the diagnosis of NAFLD.

In conclusion, ALT levels of >19 IU/l or TG levels of >101 mg/dl were useful markers for the screening of NAFLD. However, a stronger marker for the diagnosis of NAFLD was the combination of ALT levels of >19 IU/l and TG levels of >101 mg/dl.

## Figures and Tables

**Table I:** Comparison of variables between the non-NAFLD and NAFLD patients.

Variables	NF group	F group	P-value
Age (years)	67.7±1.5	66.0±1.5	0.4049
ALP (IU/l)	254.9±18.1	278.9±15.1	0.3126
AST (IU/l)	12.8±3.2	38.5±2.9	0.0077
ALT (IU/l)	20.6±4.0	44.5±3.6	<0.0001
γ-GTP (IU/l)	52.8±17.0	76.7±14.0	0.2815
LDH (IU/l)	198.7±5.6	202.2±7.3	0.6948
HDL (mg/dl)	63.2±2.2	54.0±2.3	0.0046
LDL (mg/dl)	109.8±3.3	121.9±3.5	0.0126
TG (mg/dl)	96.1±6.8	163.1±7.4	<0.0001
T-chol (mg/dl)	193.1±4.4	211.7±4.6	0.0042

Data are expressed as the mean ± standard error. NAFLD, non-alcoholic fatty liver disease; ALP, alkaline phosphatase; AST, aspartate aminotransferase; ALT, alanine aminotransferase; γ-GTP: γ-glutamyl transpeptidase; LDH, lactate dehydrogenase; HDL, high-density lipoprotein cholesterol; LDL, low-density lipoprotein cholesterol; TG, triglycerides; T-chol, total cholesterol; NF, non-NAFLD group; F, NAFLD group.

**Table II:** AUC, thresholds, sensitivity and specificity values for the variables.

Variables	AUC	Threshold	Sensitivity (%)	Specificity (%)
Age (years)	0.566	72.0	75.8	41.4
ALP (IU/l)	0.550	300	40.0	81.0
AST (IU/l)	0.654	30.0	53.9	76.5
ALT (IU/l)	0.743	19.0	80.0	63.5
γ-GTP (IU/l)	0.698	48.0	53.1	84.3
LDH (IU/l)	0.536	194	58.3	56.7
HDL (mg/dl)	0.635	61.1	71.2	54.2
LDL (mg/dl)	0.632	118	61.3	62.5
TG (mg/dl)	0.778	101	78.4	64.3
T-chol (mg/dl)	0.638	192	77.5	48.3

ALP, alkaline phosphatase; AST, aspartate aminotransferase; ALT, alanine aminotransferase; γ-GTP, γ-glutamyl transpeptidase; LDH, lactate dehydrogenase; HDL, high-density lipoprotein cholesterol; LDL, low-density lipoprotein cholesterol; TG, triglycerides; T-chol, total cholesterol; AUC, area under the curve.

**Table III:** Diagnosis of fatty liver (number of patients).

Subgroup	F group	NF group	Total
Positive	54	18	72
Negative	26	76	102
Total	80	94	174

Patients in the positive group had ALT levels of >19 IU/l and TG levels of >101 mg/dl, while patients in the negative group had levels lower than the stated thresholds. ALT, alanine aminotransferase; TG, triglyceride; NF, non-NAFLD group; F, NAFLD group; NAFLD, non-alcoholic fatty liver disease.
